# Hydroxychloroquine for treatment of COVID-19 patients: a systematic review and meta-analysis of randomized controlled trials

**DOI:** 10.31744/einstein_journal/2022RW0045

**Published:** 2022-11-18

**Authors:** Vinícius Ynoe de Moraes, Alexandre Rodrigues Marra, Leandro Luongo Matos, Ary Serpa, Luiz Vicente Rizzo, Miguel Cendoroglo, Mario Lenza

**Affiliations:** 1 Hospital Israelita Albert Einstein São Paulo SP Brazil Hospital Israelita Albert Einstein, São Paulo, SP, Brazil.

**Keywords:** Hydroxychloroquine, COVID-19, Coronavirus infections, SARS-CoV-2, Drug-related side effects and adverse reactions, Therapeutics, Disease prevention

## Abstract

**Objective:**

We performed a systematic review of the literature and meta-analysis on the efficacy and safety of hydroxychloroquine to treat COVID-19 patients.

**Methods:**

We searched the Cochrane Central Register of Controlled Trials (CENTRAL), MEDLINE, EMBASE, and LILACS (January 2019 to March 2021) for patients aged 18 years or older, who had COVID-19 and were treated with hydroxychloroquine *versus* placebo or standard of care. We also searched the WHO Clinical Trials Registry for ongoing and recently completed studies, and the reference lists of selected articles and reviews for possible relevant studies, with no restrictions regarding language or publication status. Random-effects models were used to obtain pooled mean differences of treatment effect on mortality, and serious adverse effects between hydroxychloroquine and the Control Group (standard of care or placebo); heterogeneity was assessed using the I^2^ and the Cochran´s Q statistic.

**Results:**

Nine studies met the inclusion criteria and were included in the meta-analysis. There was no significant difference in mortality rate between patients treated with hydroxychloroquine compared to standard of care or placebo (16.7% *versus* 18.5%; pooled risk ratio 1.09; 95% confidence interval: 0.99-1.19). Also, the rate of serious adverse effects was similar between both Groups, Hydroxychloroquine and Control (3.7% *versus* 2.9%; pooled risk ratio 1.22; 95% confidence interval: 0.76-1.96).

**Conclusion:**

Hydroxychloroquine is not efficacious in reducing mortality of COVID-19 patients.

**Prospero database registration:**

(www.crd.york.ac.uk/prospero) under number CRD42020197070.

## INTRODUCTION

Hydroxychloroquine (HCQ) has received worldwide attention as a potential treatment for coronavirus disease 2019 (COVID-19) because of positive results from small studies.^(1,2)^ Since then, HCQ, combined or not with azithromycin, has been considered as a possible therapeutic agent for patients with COVID-19.^(2,3)^ Although we have been facing the challenges of this pandemic for a long time, there are very few specific treatments for COVID-19 patients.^(4,5)^ The vast majority of studies assessing treatments for these patients were small clinical studies, which were not acceptable, even in a pandemic, due to their design and characteristics (*e.g.*, non-randomized, underpowered, and/or open label). In addition, the medical principle “first do not harm” should be one of the first principles in any clinical study.^(6)^

There are several reports and studies on the potential effect of HCQ on inhibiting the action of various viruses, such as other coronaviruses (SARS-CoV-1, MERS-CoV), Ebola virus, HIV, influenza virus (H1N1) and hepatitis B and C viruses.^(7)^ However, there is a huge difference between what happens *in vitro* and *in vivo.*^(8)^ Moreover, the association of HCQ and other antimicrobial therapy, and the potential adverse events, have not been fully understood yet. Although we recognized that the topic has already been vastly explored in the literature, our approach is of merit as it was previously delineated in the early days of the COVID-19 pandemic (from a priori developed protocol), followed Cochrane collaboration standards for conducting systematic reviews and also included experts in the topic for the assessment of the studies and data analysis.

## OBJECTIVE

We aimed to perform a systematic review of the literature and a meta-analysis and evaluate the effects of hydroxychloroquine prescription to treat adult COVID-19 patients, considering mortality and prevention of serious adverse events.

## METHODS

The systematic review of the literature was conducted in line with PRISMA guidelines and Cochrane handbook.^(9,10)^ It included all randomized controlled trials (RCTs) published, which assessed COVID-19 patients aged 18 years or older, who were treated with chloroquine or HCQ, at any dose, and compared to a Control Group that received other standard of care treatment, supportive treatment or placebo. We excluded prevention or post-exposure prophylaxis studies.

### Search strategy

We searched the Cochrane Central Register of Controlled Trials (CENTRAL; in The Cochrane Library current issue), MEDLINE, EMBASE, and LILACS (January 2019 to March 2021). We also searched the WHO Clinical Trials Registry for ongoing and recently completed studies, and the reference lists of selected articles and reviews for possible relevant studies, with no restrictions regarding language or publication status.

In MEDLINE (PubMed), we combined the subject-specific search (((“coronavirus”[mesh terms]) or (coronavirus*[title/abstract] or coronovirus*[title/abstract] or coronavirinae*[title/abstract] or coronavirus*[title/abstract] or coronovirus*[title/abstract] or wuhan*[title/abstract] or hubei*[title/abstract] or huaian[title/abstract] or “2019-ncov”[title/abstract] or 2019ncov[title/ab- stract] or ncov2019[title/abstract] or “ncov-2019”[title/abstract] or “covid-19”[title/abstract] or covid19[title/abstract] or “hcov-19”[title/abstract] or hcov19[title/abstract] or cov[title/abstract] or “2019 novel*”[title/abstract] or ncov[title/abstract] or “n-cov”[title/abstract] or “sars-cov-2”[title/ abstract] or “sarscov-2”[title/abstract] or “sarscov2”[title/abstract] or “sars-cov2”[title/abstract] or “sars-cov-19”[title/abstract] or ncorona*[title/abstract])) AND (((((((Hydroxychloroquine[MeSH Terms]) OR (Chloroquine[MeSH Terms])) OR (chloroquin*[Title/Abstract])) OR (Hydroxychloro- quin*[Title/Abstract])) OR (Oxychloroquin*[Title/Abstract])) OR (antimalaria*[Title/Abstract])) OR (anti-malaria*[Title/Abstract]))). Search strategies were adapted to The Cochrane Library (Wiley InterScience), EMBASE (Ovid Web), and LILACS.

### Selection of studies

Two authors independently identified and selected potentially eligible studies for inclusion in the review. Any disagreements were resolved by discussion and consensus. A third author was included in the discussion, if needed. The review authors were not blinded to the journal or authors.

### Data extraction

Two authors independently extracted the following data using a specific extraction form: characteristics of the study including study design, duration of the study, if the protocol was published before recruitment of patients, funding sources, and details of trial registration; characteristics of the study including place of study, number of participants assigned, number of participants assessed, inclusion criteria, exclusion criteria and age; characteristics of the study interventions including timing and type of intervention and control, and any co-interventions; characteristics of the study outcomes including the length of follow-up, loss to follow-up, and outcome measures; and the methodological domains looking for risk of bias. Any disagreements were resolved by discussion.

The risk of bias of the included studies was assessed by two independent authors. As recommended by The Cochrane Collaboration Risk of Bias tool,^(11)^ we assessed the following domains: random sequence generation; allocation concealment; blinding of participants and personnel; blinding of outcome assessment; incomplete outcome data; selective reporting; and other bias. Each of these criteria was evaluated using low risk of bias; high risk of bias; and unclear (either lack of information or uncertainty over potential for bias). Disagreements between authors regarding the risk of bias for the domains were resolved by discussion.

### Outcomes

The primary outcome was all-cause mortality. As second outcome, we evaluated serious adverse events of HCQ treatment (life-threatening or requiring hospitalization or adverse events that resulted in discontinuation of treatment). Other outcomes could not be assessed due to substantial heterogeneity between measurements and outcomes of the included studies.

### Statistical analysis

We combined the results of the included trials by performing a meta-analysis, using the Review Manager version 5.0 (The Cochrane Collaboration), and a p<0.05 was considered significant.^(12)^ For rate comparisons, we calculated the risk ratio (RR) with a 95% confidence interval (95%CI) for individual studies. We pooled similar studies using a random-effects model, according to the Mantel-Haenszel method for estimating the RR and its 95%CI, and pooled data are shown in forest plots.

The unit of randomization for all trials included was the individual participant of study. There were no unit of analysis issues when considering cluster-randomized trials. Where appropriate, problems of unit of analysis with multiple reporting of outcomes, such as different follow-up times were solved by presenting these separately.

The presence of heterogeneity among the studies was estimated by the Cochran´s Q statistic and measured by the I^2^ value, and heterogeneity was considered present for I^2^>50%. Data from the systematic review were grouped, and the weighted average was calculated as the studies’ summary measure. Funnel plots were obtained to estimate the publication bias.

### Assessment of quality of evidence and ‘Summary of findings’ table

The GRADE approach was used to assess the quality of evidence related to two outcomes - mortality and serious adverse events.^(13)^ Quality of evidence was categorized as ‘high’, ‘moderate’, ‘low’, or ‘very low’, depending on the presence and extent of five factors: risk of bias, inconsistency of effect, indirectness, imprecision, and publication bias.

The main results of the use of HCQ to treat participants with COVID-19 are presented in a ‘Summary of findings’ table, which provides key information concerning quality of evidence, magnitude of effect of the interventions examined, and the sum of available data on the main outcomes.

## RESULTS

### Results of the search

**Figure 1:**
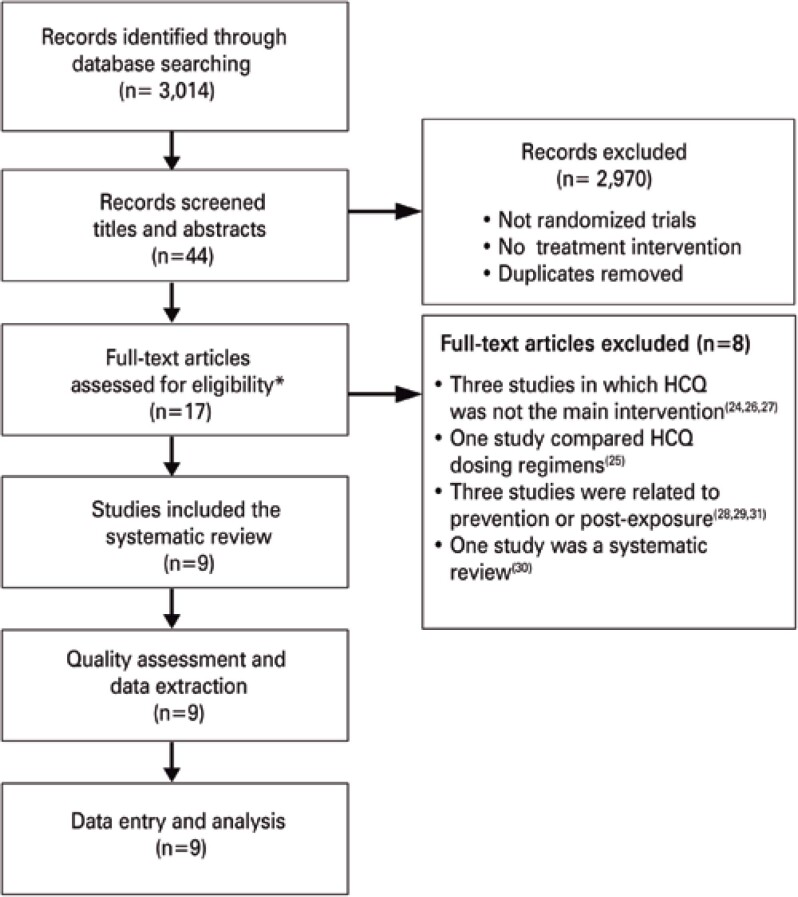
Flowchart of article selection

The searches for this review identified a total of 3,014 articles for analysis, and 44 reports of potentially eligible studies, for which we obtained full reports, whenever possible. Of these, nine studies^(14–22)^ were included, and eight, excluded.^(23–30)^ The flowchart of article selection is available in figure 1.

* retrieved articles.

The number of subjects per study ranged from 19 to 1,561 participants in the Treatment Group, and from 11 to 3,155 in the Control Group. All studies included evaluated only HCQ in the intervention arm, as described in table 1.

**Table 1:** Main characteristics of the included studies

Study ID	Country	Site	Drug	HCQ regimens	Control	Age (years)	Subjects on HCQ	Subjects on control	Diagnosis	RT-PCR	Covid, mild	Covid, moderate	Covid, severe	Declaration of funding
Abd-Elsalam et al.,^(14)^	Egypt	Hospital	HCQ	800mg (1 day), maintenance 400mg/day (15 days)	Standard of care, oseltamivir (if needed)	18+	97	97	Oropharyngeal swab	Yes	Yes	Yes	Yes	No
Cavalcanti et al.,^(15)^	Brazil	Hospital	HCQ	800mg/day (7 days)	Standard of care	18+	221	227	Oropharyngeal swab	Yes	Yes	Yes	No	Yes
Chen et al.,^(16)^	China (Taiwan)	Hospital	HCQ	1000mg (1 day), maintence 500mg/day (9 days)	Standard of care	18+	19	11	Oropharyngeal swab	Yes	Yes	Yes	No	Yes
Lyngbakken et al.,^(17)^	Norway	Hospital	HCQ	400mg/day for 7 days	Standard of care	18+	26	25	Oropharyngeal swab	Yes	No	Yes	No	Yes
Pan et al.^(18)^	WHO (30 countries)	Hospital	HCQ	800mg (1 day), 800mg after 6 hours, maintenance 800mg/day (10 days)	Standard of care	18+	954	906	Any	Yes	No	Yes	Yes	Yes
RECOVERY et al.,^(19)^	UK	Hospital	HCQ	1600mg/day, 800mg/day (for 10 days) or discharge	Standard of care	Any	1,561	3,155	Oropharyngeal swab	Yes	Yes	Yes	Yes	Yes
Self et al.,^(20)^	USA	Hospital Non-Hospital	HCQ	800mg (1 day), maintenance 400mg/day (4 days)	Placebo	18+	242	237	Oropharyngeal swab	Yes	Yes	Yes	Yes	Yes
Skipper et al.,^(21)^	USA Canada	Non-hospital	HCQ	800mg+600mg (1 day), maintenance 600mg/day (4 days)	Placebo	18+	244	247	Oropharyngeal swab, or symptoms and exposure	Yes	Yes	No	No	Yes
Tang et al.,^(22)^	China	Hospital	HCQ	1200mg/day (3 days), maintenance 800mg/day	Standard of care	18+	75	75	Oropharyngeal sample	Yes	Yes	Yes	Yes	No

WHO: World Health Organization; UK: United Kingdom; USA: United Staes of America; HCQ: hydroxychloroquine; RT-PCR: reverse transcription-polymerase chain reaction.

### Quality assessment

**Figure 2:**
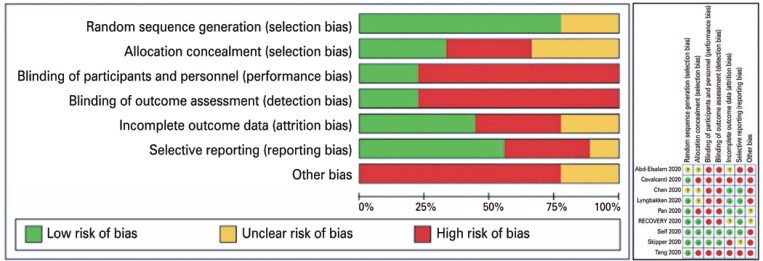
Summarized and individual risk of bias assessment of the included studies

Overall quality of the trials was compatible with COVID-19 pandemic scenario and most fulfilled the expected standards. One important caveat to be pinpointed is the fact that most of the trials were designed to produce a more pragmatic than explanatory evidence (real world evidence). Trials failed most at blinding participants and personnel, and we had some special concerns regarding some underpowered trials, which we downgraded to the “other bias” section. Some other concerns are related to lack of standardization to define adverse effects and their stratification. These criteria had substantially varied between trials. Figure 2 summarizes the main aspects regarding risk or quality assessment.

### Effects of interventions

Table 2 shows the summary of findings for the main comparisons of HCQ to treat participants with COVID-19. The primary comparison in this review was HCQ *versus* standard of care in management of COVID-19 patients. We presented our results for two endpoints: mortality and serious adverse events.

**Table 2:** Hydroxychloroquine compared to standard of care for patients with COVID-19

**Patient or population:** patients with COVID-19 **Setting:** Hospital / outpatient clinic **Intervention:** hydroxychloroquine **Comparison:** standard of care						
Outcomes	Anticipated absolute effects* (95%CI)		Relative effects (95%CI)	Number of participants (studies)	Certainty of the evidence (GRADE)	Comments
	Risk with standard of care	Risk with hydroxychloroquine				
Mortality	185 per 1,000	202 per 1,000 (183 to 220)	RR 1.09 (0.99 to 1.19)	8303 (9 RCTs)	⊕⊕◯◯ Low^a^,^b^	The evidence suggests that hydroxychloroquine results in little to no difference in mortality
Serious adverse events	29 per 1,000	35 per 1,000 (22 to 57)	RR 1.22 (0.76 to 1.96)	6242 (6 RCTs)	⊕◯◯◯ Very low^a^,^b^,^c^,^d^	The evidence is very uncertain about the effect of hydroxychloroquine on severe adverse events

* The risk in the intervention group (and its 95% confidence interval) is based on the assumed risk in the comparison group and the relative effect of the intervention (and its 95%CI).

95%CI: 95% confidence interval; RR: risk ratio; RTC: randomized controlled trials; HCQ: hydroxychloroquine.

GRADE Working Group grades of evidence.

High certainty: we are very confident that the true effect lies close to that of the estimate of the effect.

Moderate certainty: we are moderately confident in the estimate: the true effects is likely to be close to the estimate of the effect, but there is a possibility that it is substantially different.

Low certainty: our confidence in the effect estimate is limited: the true effect may be substantially different from the estimate of the effect.

Very low certainty: we have very little confidence in the effect estimate: the true effect is likely to be substantially different from the estimate of effect.

Explanations

a Issues regarding allocation concealment and incomplete data (attrition bias).

b Treatment protocols, such as drug dosage and duration differed among trials. Participants combined in-hospital and outpatient use of HCQ.

c Issues regarding to allocation concealment and incomplete data (attrition bias). Criteria for determining adverse effects different among trials.

d Wider confidence interval demonstrates either small benefit or very serious harm.

### Mortality

**Figure 3:**
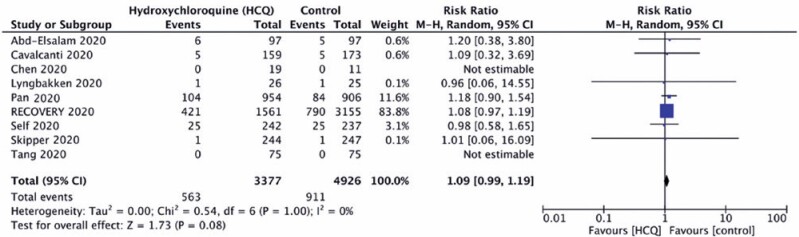
Forest plot demonstrating no effect of hydroxychloroquine on mortality reduction

**Figure 4:**
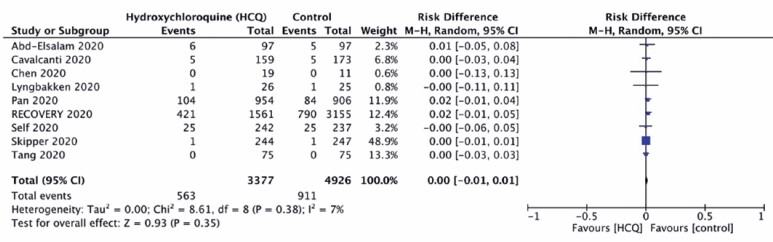
Forest plot demonstrating no effect of hydroxychloroquine on mortality reduction (risk ratio)

Hydroxychloroquine did not significantly reduce the mortality rate of COVID-19 when compared to standard of care (Control Group) (16.7% *versus* 18.5%; RR 1.09; 95%CI: 0.99-1.19; risk difference=0.00; 95%CI: -0.01-0.01), with no relevant heterogeneity across the studies, as demonstrated in figure 3 and 4.

M-H: Mantel-Haenszel; 95%CI: 95% confidence interval.

M-H: Mantel-Haenszel; 95%CI: 95% confidence interval.

### Serious adverse events

**Figure 5:**
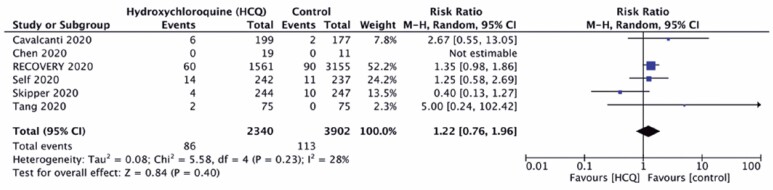
Forest plot demonstrating no impact of hydroxychloroquine on serious adverse events

The rate of serious adverse effects was similar in both Groups HCQ and Control (3.7% *versus* 2.9%; RR 1.22; 95%CI: 0.76-1.96), with low heterogeneity across the studies (I^2^=28%), as shown in figure 5.

M-H: Mantel-Haenszel; 95%CI: 95% confidence interval.

### Reporting bias

**Figure 6:**
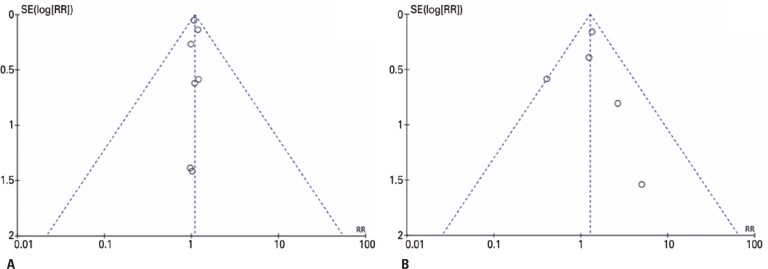
Funnel plots evaluating publication bias for mortality (A) and serious adverse events (B) analyses

A robust analysis of potential publication bias was not possible due to the small number of studies published in the specific literature. However, a potential publication bias for mortality was not identified when evaluating the funnel plot (Figure 6). Besides the relation with serious adverse events (Figure 6) could not be well established.

## DISCUSSION

In the present systematic review of the literature and meta-analysis including nine randomized clinical trials, and 8,419 COVID-19 patients, there was no significant reduction in mortality when HCQ was used as treatment. Moreover, the drug was not related to an increase in serious adverse events. Not all studies provided evidence for all outcomes, and the quality of evidence for important outcomes was low for mortality, and very low for adverse events.

A growing number of hospitals reconsidered treating COVID-19 patients with HCQ after the first publications, in mid-2020´s.^(19)^ However, HCQ has received much attention and been very widely employed to treat COVID-19 patients, despite the absence of any good evidence. Altogether, these findings support the absence of evidence on the use of HCQ to treat COVID-19 patients. Similarly, other therapeutical options considered should be investigated through well-powered properly designed RCTs.

In clinical trials, the action of the drug studied is expected to bring some benefits for human beings, in terms of clinical outcomes. In other words, we need a drug that allows for longer survival, and avoid deaths. Furthermore, no adverse events are desired; however, if that is not possible, then let them be the fewer and the least harmful possible. All of these clinical outcomes should be evaluated in a clinical study. For drug evaluation, randomized studies are required. The good news is there are many ongoing studies on COVID-19. Based on this approach, our systematic review and meta-analysis only included RCTs. We also opted not to include non-randomized, quasi-experimental studies, which aim to demonstrate causality between an intervention and an outcome, and encompass a broad range of non-randomized intervention studies. These designs are frequently used when it is not logistically feasible or ethical to conduct a RCT.^(31)^ However, this is not the case for the COVID-19 pandemic. For COVID-19 patients, there is still no treatment capable of acting on the severe acute respiratory syndrome coronavirus 2 (SARS-CoV-2) virus, and that is why we chose to seek the best evidence, by using only RCTs in our meta-analysis. We also conducted a systematic review of the literature after one year of the COVID-19 pandemic, to have enough time for RCTs to be published. Previous reported systematic reviews and meta-analyses evaluated safety of HCQ in COVID-19, and focused only on adverse events,^(32)^ or included just a small portion of these RCTs,^(33)^ or also included unpublished clinical trial.^(34)^ Differently from them, we focused on investigating studies that evaluated mortality as outcome, reported severe adverse events associated to COVID-19.

The literature presents some misleading information on *in vitro* data and clinical trials. Hydroxychloroquine and chloroquine were shown to prevent viral infection in cell-culture systems; nonetheless, clinical trials in humans did not detect a significant improvement in COVID-19 patients treated with these drugs. Studies reported that HCQ and chloroquine slightly decreased the viability of Vero, TMPRSS2-expressing Vero and Calu-3 cells when introduced at the highest concentration. Hydroxychloroquine and chloroquine could block S-driven entry, but this inhibition is cell-line-dependent, and efficient inhibition has not been observed in TMPRSS2+ lung cells.^(35,36)^

Our analysis has some limitations. The interpretation of these findings requires caution due to substantial differences among the studies included. In addition, the population of these studies is heterogeneous, comprising hospitalized and non-hospitalized patients. It is important to emphasize this aspect because when we searched the number of deaths due to COVID-19, we attributed it as mortality in our meta-analysis but with no real-time definition of it. For analysis of serious adverse events, each study adopted a different definition. Moreover, the HCQ dose criteria were different among studies. Most of them considered a loading dose, and maintenance doses varying from 400-800mg/day; and total treatment time was also diverse. Considering sample size, the RECOVERY study^(19)^ had a large sample (weight of more than 80% in the compilation of our meta-analysis for mortality), (weight of more than 10%);^(18)^ both studies indicated no mortality benefit for HCQ use against COVID-19. Even including only RCTs in our meta-analysis, just two studies were double-blinded.^(20,21)^ Double-blinded trials are thought to produce objective results since the expectations of researchers and participants about the experimental treatment, such as HCQ, do not affect the outcome. Although it does not reflect the real-life circumstances, and this may be one of the main reasons for most of these studies not adopting double-blind methods. Another important point was publication bias could not be properly addressed, since the number of included studies was small.

In the same scope of our study, one Cochrane Collaboration review reported similar results, and the authors highlighted the rates of adverse effects, pointing out that most of them were not serious.^(37)^ New studies, such as the COPE-Coalition V, which recruited 1,372 non-hospitalized patients and assessed the risk of hospitalization, produced similar findings and are unlikely to change the magnitude and direction of our findings.^(38)^ From a global perspective, a lot of criticism was pointed out in trials conducted in the worst moments of the COVID-19 pandemic, which made researchers and policy advisors aware of actions to take in next pandemics.^(39)^

## CONCLUSION

In conclusion, our systematic review and meta-analysis, exclusively with published randomized controlled trials, found treatment with hydroxychloroquine is not efficacious to reduce mortality of COVID-19 patients.

## References

[1] Devaux CA, Rolain JM, Colson P, Raoult D (2020). New insights on the antiviral effects of chloroquine against coronavirus: what to expect for COVID-19?. Int J Antimicrob Agents.

[2] Meo SA, Klonoff DC, Akram J (2020). Efficacy of chloroquine and hydroxychloroquine in the treatment of COVID-19. Eur Rev Med Pharmacol Sci.

[3] Fan J, Zhang X, Liu J, Yang Y, Zheng N, Liu Q (2020). Connecting hydroxychloroquine in vitro antiviral activity to in vivo concentration for prediction of antiviral effect: a critical step in treating patients with coronavirus disease 2019. Clin Infect Dis.

[4] Beigel JH, Tomashek KM, Dodd LE, Mehta AK, Zingman BS, Kalil AC, Hohmann E, Chu HY, Luetkemeyer A, Kline S, Lopez de Castilla D, Finberg RW, Dierberg K, Tapson V, Hsieh L, Patterson TF, Paredes R, Sweeney DA, Short WR, Touloumi G, Lye DC, Ohmagari N, Oh MD, Ruiz-Palacios GM, Benfield T, Fätkenheuer G, Kortepeter MG, Atmar RL, Creech CB, Lundgren J, Babiker AG, Pett S, Neaton JD, Burgess TH, Bonnett T, Green M, Makowski M, Osinusi A, Nayak S, Lane HC (2020). ACTT-1 Study Group Members. Remdesivir for the treatment of Covid-19 - final report. N Engl J Med.

[5] Hung IF, Lung KC, Tso EY, Liu R, Chung TW, Chu MY (2020). Triple combination of interferon beta-1b, lopinavir-ritonavir, and ribavirin in the treatment of patients admitted to hospital with COVID-19: an open-label, randomised, phase 2 trial. Lancet.

[6] Kohn LT, Corrigan JM, Donaldson MS, Institute of Medicine (US) Committee on Quality of Health Care in America (2000). To err is human: building a safer health system.

[7] Al-Bari MA (2017). Targeting endosomal acidification by chloroquine analogs as a promising strategy for the treatment of emerging viral diseases. Pharmacol Res Perspect.

[8] Marcolino VA, Pimentel TC, Barão CE (2020). What to expect from different drugs used in the treatment of COVID-19: a study on applications and in vivo and in vitro results. Eur J Pharmacol.

[9] Moher D, Liberati A, Tetzlaff J, Altman DG (2010). PRISMA Group. Preferred reporting items for systematic reviews and meta-analyses: the PRISMA statement. Int J Surg.

[10] Higgins JP, Thomas J, Chandler J, Cumpston M, Li T, Page MJ (2019). Cochrane Handbook for Systematic Reviews of Interventions version 6.0 (updated July 2019).

[11] Higgins JP, Savović J, Page MJ, Elbers RG, Sterne JA, Higgins JP, Thomas J, Chandler J, Cumpston M, Li T, Page MJ (2019). Cochrane Handbook for Systematic Reviews of Interventions version 6.0 (updated July 2019).

[12] Cochrane training (2020). ReviewManager (RevMan) is Cochrane’s bespoke software for writing Cochrane Reviews.

[13] Schünemann HJ, Higgins JP, Vist GE, Glasziou P, Akl EA, Skoetz N, Higgins JP, Thomas J, Chandler J, Cumpston M, Li T, Page MJ (2022). Cochrane Handbook for Systematic Reviews of Interventions.

[14] Abd-Elsalam S, Esmail ES, Khalaf M, Abdo EF, Medhat MA, Abd El Ghafar MS (2020). Hydroxychloroquine in the treatment of COVID-19: a multicenter randomized controlled study. Am J Trop Med Hyg.

[15] Cavalcanti AB, Zampieri FG, Rosa RG, Azevedo LC, Veiga VC, Avezum A, Damiani LP, Marcadenti A, Kawano-Dourado L, Lisboa T, Junqueira DL, de Barros E, Silva PG, Tramujas L, Abreu-Silva EO, Laranjeira LN, Soares AT, Echenique LS, Pereira AJ, Freitas FG, Gebara OC, Dantas VC, Furtado RH, Milan EP, Golin NA, Cardoso FF, Maia IS, Hoffmann CR, Kormann AP, Amazonas RB, Bocchi de Oliveira MF, Serpa-Neto A, Falavigna M, Lopes RD, Machado FR, Berwanger O (2020). Coalition Covid-19 Brazil I Investigators. Hydroxychloroquine with or without azithromycin in mild-to-moderate Covid-19. N Engl J Med.

[16] Chen L, Zhang Z, Fu J, Feng Z, Zhang S, Han Q (2020). Efficacy and safety of chloroquine or hydroxychloroquine in moderate type of COVID-19: a prospective open-label randomized controlled study. medRxiv.

[17] Lyngbakken MN, Berdal JE, Eskesen A, Kvale D, Olsen IC, Rueegg CS (2020). A pragmatic randomized controlled trial reports lack of efficacy of hydroxychloroquine on coronavirus disease 2019 viral kinetics. Nat Commun.

[18] Pan H, Peto R, Henao-Restrepo AM, Preziosi MP, Sathiyamoorthy V, Abdool Karim Q, Alejandria MM, Hernández García C, Kieny MP, Malekzadeh R, Murthy S, Reddy KS, Roses Periago M, Abi Hanna P, Ader F, Al-Bader AM, Alhasawi A, Allum E, Alotaibi A, Alvarez-Moreno CA, Appadoo S, Asiri A, Aukrust P, Barratt-Due A, Bellani S, Branca M, Cappel-Porter HB, Cerrato N, Chow TS, Como N, Eustace J, García PJ, Godbole S, Gotuzzo E, Griskevicius L, Hamra R, Hassan M, Hassany M, Hutton D, Irmansyah I, Jancoriene L, Kirwan J, Kumar S, Lennon P, Lopardo G, Lydon P, Magrini N, Maguire T, Manevska S, Manuel O, McGinty S, Medina MT, Mesa Rubio ML, Miranda-Montoya MC, Nel J, Nunes EP, Perola M, Portolés A, Rasmin MR, Raza A, Rees H, Reges PP, Rogers CA, Salami K, Salvadori MI, Sinani N, Sterne JA, Stevanovikj M, Tacconelli E, Tikkinen KA, Trelle S, Zaid H, Røttingen JA, Swaminathan S, WHO Solidarity Trial Consortium (2020). Repurposed antiviral drugs for Covid-19 - interim WHO Solidarity Trial results. N Engl J Med.

[19] Horby P, Mafham M, Linsell L, Bell JL, Staplin N, Emberson JR, Wiselka M, Ustianowski A, Elmahi E, Prudon B, Whitehouse T, Felton T, Williams J, Faccenda J, Underwood J, Baillie JK, Chappell LC, Faust SN, Jaki T, Jeffery K, Lim WS, Montgomery A, Rowan K, Tarning J, Watson JA, White NJ, Juszczak E, Haynes R, Landray MJ, RECOVERY Collaborative Group (2020). Effect of hydroxychloroquine in hospitalized patients with Covid-19. N Engl J Med.

[20] Self WH, Semler MW, Leither LM, Casey JD, Angus DC, Brower RG (2020). Effect of hydroxychloroquine on clinical status at 14 days in hospitalized patients with COVID-19: a randomized clinical trial. JAMA.

[21] Skipper CP, Pastick KA, Engen NW, Bangdiwala AS, Abassi M, Lofgren SM (2020). Hydroxychloroquine in nonhospitalized adults with early COVID-19: a randomized trial. Ann Intern Med.

[22] Tang W, Cao Z, Han M, Wang Z, Chen J, Sun W (2020). Hydroxychloroquine in patients with mainly mild to moderate coronavirus disease 2019: open label, randomised controlled trial. BMJ.

[23] Furtado RH, Berwanger O, Fonseca HA, Corrêa TD, Ferraz LR, Lapa MG, Zampieri FG, Veiga VC, Azevedo LC, Rosa RG, Lopes RD, Avezum A, Manoel AL, Piza FM, Martins PA, Lisboa TC, Pereira AJ, Olivato GB, Dantas VC, Milan EP, Gebara OC, Amazonas RB, Oliveira MB, Soares RV, Moia DD, Piano LP, Castilho K, Momesso RG, Schettino GP, Rizzo LV, Neto AS, Machado FR, Cavalcanti AB (2020). COALITION COVID-19 Brazil II Investigators. Azithromycin in addition to standard of care versus standard of care alone in the treatment of patients admitted to the hospital with severe COVID-19 in Brazil (COALITION II): a randomised clinical trial. Lancet.

[24] Borba MG, Val FF, Sampaio VS, Alexandre MA, Melo GC, Brito M, Mourão MP, Brito-Sousa JD, Baía-da-Silva D, Guerra MV, Hajjar LA, Pinto RC, Balieiro AA, Pacheco AG, Santos JD, Naveca FG, Xavier MS, Siqueira AM, Schwarzbold A, Croda J, Nogueira ML, Romero GA, Bassat Q, Fontes CJ, Albuquerque BC, Daniel-Ribeiro CT, Monteiro WM, Lacerda MV (2020). CloroCovid-19 Team. Effect of high vs low doses of chloroquine diphosphate as adjunctive therapy for patients hospitalized with severe acute respiratory syndrome coronavirus 2 (SARS-CoV-2) infection: a randomized clinical trial. JAMA Netw Open.

[25] Sekhavati E, Jafari F, SeyedAlinaghi S, Jamalimoghadamsiahkali S, Sadr S, Tabarestani M (2020). Safety and effectiveness of azithromycin in patients with COVID-19: an open-label randomised trial. Int J Antimicrob Agents.

[26] Scarsi M, Piantoni S, Colombo E, Airó P, Richini D, Miclini M (2020). Association between treatment with colchicine and improved survival in a single-centre cohort of adult hospitalised patients with COVID-19 pneumonia and acute respiratory distress syndrome. Ann Rheum Dis.

[27] Mitjà O, Corbacho-Monné M, Ubals M, Tebé C, Peñafiel J, Tobias A (2021). Hydroxychloroquine for early treatment of adults with mild coronavirus disease 2019: a randomized, controlled trial. Clin Infect Dis.

[28] Boulware DR, Pullen MF, Bangdiwala AS, Pastick KA, Lofgren SM, Okafor EC (2020). A randomized trial of hydroxychloroquine as postexposure prophylaxis for Covid-19. N Engl J Med.

[29] Pastick KA, Okafor EC, Wang F, Lofgren SM, Skipper CP, Nicol MR (2020). Review: Hydroxychloroquine and chloroquine for treatment of SARS-CoV-2 (COVID-19). Open Forum Infect Dis.

[30] Rajasingham R, Bangdiwala AS, Nicol MR, Skipper CP, Pastick KA, Axelrod ML, Pullen MF, Nascene AA, Williams DA, Engen NW, Okafor EC, Rini BI, Mayer IA, McDonald EG, Lee TC, Li P, MacKenzie LJ, Balko JM, Dunlop SJ, Hullsiek KH, Boulware DR, Lofgren SM (2021). COVID PREP team. Hydroxychloroquine as pre-exposure prophylaxis for coronavirus disease 2019 (COVID-19) in healthcare workers: a randomized trial. Clin Infect Dis.

[31] Harris AD, Lautenbach E, Perencevich E (2005). A systematic review of quasi-experimental study designs in the fields of infection control and antibiotic resistance. Clin Infect Dis.

[32] Chen C, Pan K, Wu B, Li X, Chen Z, Xu Q (2021). Safety of hydroxychloroquine in COVID-19 and other diseases: a systematic review and meta-analysis of 53 randomized trials. Eur J Clin Pharmacol.

[33] Siemieniuk RA, Bartoszko JJ, Zeraatkar D, Kum E, Qasim A, Martinez JP (2020). Drug treatments for covid-19: living systematic review and network meta-analysis. BMJ.

[34] Axfors C, Schmitt AM, Janiaud P, Van’t Hooft J, Abd-Elsalam S, Abdo EF (2021). Author Correction: Mortality outcomes with hydroxychloroquine and chloroquine in COVID-19 from an international collaborative meta-analysis of randomized trials. Nat Commun.

[35] Ou T, Mou H, Zhang L, Ojha A, Choe H, Farzan M (2021). Hydroxychloroquine-mediated inhibition of SARS-CoV-2 entry is attenuated by TMPRSS2. PLoS Pathog.

[36] Hoffmann M, Mösbauer K, Hofmann-Winkler H, Kaul A, Kleine-Weber H, Krüger N (2020). Chloroquine does not inhibit infection of human lung cells with SARS-CoV-2. Nature.

[37] Singh B, Ryan H, Kredo T, Chaplin M, Fletcher T (2021). Chloroquine or hydroxychloroquine for prevention and treatment of COVID-19. Cochrane Database Syst Rev.

[38] Avezum Á, Oliveira GB, Oliveira H, Lucchetta RC, Pereira VF, Dabarian AL, D O Vieira R, Silva DV, Kormann AP, Tognon AP, De Gasperi R, Hernandes ME, Feitosa AD, Piscopo A, Souza AS, Miguel CH, Nogueira VO, Minelli C, Magalhães CC, Morejon KM, Bicudo LS, Souza GE, Gomes MA, Fo JJ, Schwarzbold AV, Zilli A, Amazonas RB, Moreira FR, Alves LB, Assis SR, Neves PD, Matuoka JY, Boszczowski I, Catarino DG, Veiga VC, Azevedo LC, Rosa RG, Lopes RD, Cavalcanti AB, Berwanger O (2022). COPE - COALITION COVID-19 Brazil V Investigators. Hydroxychloroquine versus placebo in the treatment of non-hospitalised patients with COVID-19 (COPE - Coalition V): a double-blind, multicentre, randomised, controlled trial. Lancet Reg Health Am.

[39] Schwartz IS, Boulware DR, Lee TC (2022). Hydroxychloroquine for COVID19: the curtains close on a comedy of errors. Lancet Reg Health Am.

